# BioAFMviewer software for simulation atomic force microscopy of molecular structures and conformational dynamics

**DOI:** 10.1016/j.yjsbx.2023.100086

**Published:** 2023-02-13

**Authors:** Romain Amyot, Noriyuki Kodera, Holger Flechsig

**Affiliations:** Nano Life Science Institute (WPI-NanoLSI), Kanazawa University, Kakuma-machi, Kanazawa, Ishikawa 920-1192, Japan

## Abstract

Atomic force microscopy (AFM) and high-speed scanning have significantly advanced real time observation of biomolecular dynamics, with applications ranging from single molecules to the cellular level. To facilitate the interpretation of resolution-limited imaging, post-experimental computational analysis plays an increasingly important role to understand AFM measurements. Data-driven simulation of AFM, computationally emulating experimental scanning, and automatized fitting has recently elevated the understanding of measured AFM topographies by inferring the underlying full 3D atomistic structures. Providing an interactive user-friendly interface for simulation AFM, the BioAFMviewer software has become an established tool within the Bio-AFM community, with a plethora of applications demonstrating how the obtained full atomistic information advances molecular understanding beyond topographic imaging. This graphical review illustrates the BioAFMviewer capacities and further emphasizes the importance of simulation AFM to complement experimental observations.

## Highlights

• BioAFMviewer is a stand-alone software package for data-driven interactive simulation atomic force microscopy.
• Automatized fitting allows to infer 3D atomistic biomolecular structures from resolution-limited experimental AFM images.
• Applications ranging from single proteins to filaments and protein lattices advance molecular understanding beyond measured surface AFM topographies.
• Simulation AFM experiments from molecular modeling can be correlated with high-speed AFM movies.

## 1 Introduction

Atomic force microscopy (AFM) and high-speed scanning (HS-AFM) are leading techniques to visualize biomolecular structures and monitor functional conformational dynamics under near-physiological conditions (Müller and Dufrêne, 2008, Ando et al., 2014). Directly filming molecular dynamics ranging from single proteins, functional assemblies of them, to cellular level phenomena has significantly advanced the understanding of important biological processes with interdisciplinary applications in areas of Life Science (Ando, 2022).

However, magic experiments do not exist, and AFM observations also have several limitations. Since the scanning tip probes only morphological changes within the accessible molecular surface, important motions may be missed and thus information about functionally relevant conformational couplings. Due to the tip-size being much larger than the atoms it interacts with, measured topographies do not resolve structural details on the sub-nanometer range. Furthermore, limitations in the scanning speed prevent to monitor a molecular movie of continuous conformational changes while HS-AFM records rather stroboscopic snapshots of biomolecular function.

While technological developments towards building next-generation HS-AFM with improved scanning speed are currently undertaken, intrinsic limitations inevitably remain. As a result of this, post-experimental computational analysis of AFM images is becoming increasingly important to facilitate the interpretation and understanding of measurements. For quantitative analysis of AFM topographies convenient software plugins within the ImageJ (Schindelin et al., 2012) platform are already routinely employed. Methods for automatized tracing of biomolecules and conformational analysis from AFM images have also been presented (Beton et al., 2021). Localization AFM, an image analysis method to compute high-resolution maps from AFM datasets, has been demonstrated to reveal sub-nanometer resolution details on protein surfaces (Heath et al., 2021).

Topical developments are aimed to predict 3D biomolecular structures from topographic AFM imaging. While docking of static structures into experimental topographies has been addressed previously (Scheuring et al., 2007, Trinh et al., 2012, Chaves and Pellequer, 2013, Chaves et al., 2013), more sophisticated computational methods of systematically fitting atomistic-level or coarse-grained structural templates into AFM images have been developed and evidenced to extract valuable information from scanning of protein surfaces (Amyot et al., 2022, Amyot and Flechsig, 2020, Dasgupta et al., 2020, Dasgupta et al., 2021, Niina et al., 2020, Niina et al., 2021). The applied methods rely on simulated scanning of molecular structures to produce simulated AFM images that can be correlated with experimental images. Pseudo AFM images obtained from simulated scanning have been previously used to support experimental observations (Uchihashi et al., 2011, Uchihashi et al., 2018).

Development of the BioAFMviewer with its user-friendly interactive interface and rich functionality has opened the opportunity for the broad Bio-AFM community to employ data-driven simulation AFM and facilitate the interpretation of experimental observations (Amyot et al., 2022, Amyot and Flechsig, 2020). Indeed, high-resolution equilibrium molecular structures of most proteins are either known from a combination of experiments (wwPDBconsortium, 2019) or can be predicted by the artificial intelligence AlphaFold program (Jumper et al., 2021). On the other hand, functional conformational dynamics can be obtained from multi-scale molecular modelling (Kenzaki et al., 2011, Poma et al., 2017, Flechsig and Mikhailov, 2019). The enormous amount of high-resolution protein data offers the great opportunity to better understand resolution-limited AFM scanning data.

For a plethora of applications, ranging from single molecular machines, protein filaments, to large-scale assemblies of protein lattices, simulation AFM and automatized rigid-body fitting has been demonstrated to advance the understanding of measured AFM topographic images by providing atomistic-level information (Amyot et al., 2022, Amyot and Flechsig, 2020). Simulation AFM allows to disambiguate the relative arrangement of functional domains in experimental AFM topographies, to identify the relative orientation of domains with respect to bound biomolecules (such as nucleic acids), to reconstruct molecular details of interdomain interactions, and even to identify the nucleotide-state of domains from experimental images. Applications of simulation AFM using the BioAFMviewer are rapidly increasing. The platform was used to construct atomic models of Annexin V protein lattices (Marchesi et al., 2021, Yamada et al., 2022) and to validate HS-AFM observations of aptamer-protein complexes (Biyani et al., 2022), SARS-CoV-2 spike proteins (Lim et al., 2021), actin-myosin complexes (Moretto et al., 2022, Matusovsky et al., 2022), and the NADH-cytochrome b5 reductase 3 enzyme (Ishimura et al., 2022). In a recent application, fitting of available structures was applied to assign catalytic states to measured topographies of enzyme shapes, validating kinetic analysis of HS-AFM imaging (Takeda et al., 2022).

## 2 BioAFMviewer software for data-driven simulation AFM

Of major importance to us was that the computational procedure of simulated AFM scanning, the mathematical methods for automatized fitting available biomolecular structures to experimental AFM images, and the tools for quantitative topography analysis are embedded within a highly versatile user-friendly graphical interface. Equally important was to share our developments with the broad Bio-AFM community to strengthen interdisciplinary research. The BioAFMviewer provides a complete software package which is freely available at https://www.bioafmviewer.com.

### 2.1 Live simulation atomic force microscopy

**Fig. 1:**
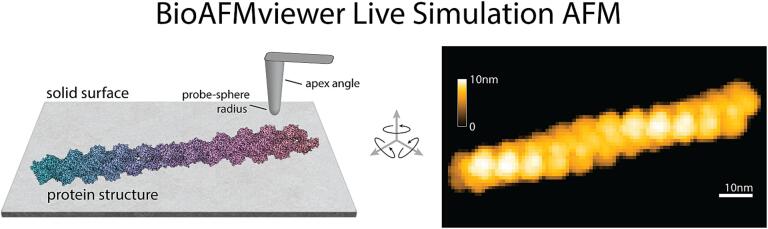
Simulation AFM emulates experimental scanning to compute a surface topographic image of a biomolecular structure which can be compared to measured AFM images. The computation is based on non-elastic collisions of a rigid conical tip with a Van-der-Waals sphere atomic model of the molecular structure placed on a solid surface, calculating a height value for each cell along the scanning grid. In the simulated AFM topographic image, heights are illustrated by a color gradient. Central to the BioAFMviewer software is an integrated molecular viewer visualizing the loaded PDB structure in any 3D orientation and synchronized computation and visualization of the corresponding simulation AFM image (*Live Simulation AFM*). The spatial resolution of scanning, tip-shape parameters, and the visual representation can be conveniently adjusted.

The idea of simulation AFM is to computationally emulate experimental scanning of biomolecules to translate their available high-resolution molecular structures into simulation AFM topographic images which can be correlated with experimentally obtained AFM images. Central to the BioAFMviewer software is an integrated molecular viewer visualizing the loaded PDB structure in any 3D orientation and synchronized computation and visualization of the corresponding simulation AFM image (Fig. 1).

### 2.2 Automatized fitting to infer 3D atomistic structures from AFM imaging

**Fig. 2:**
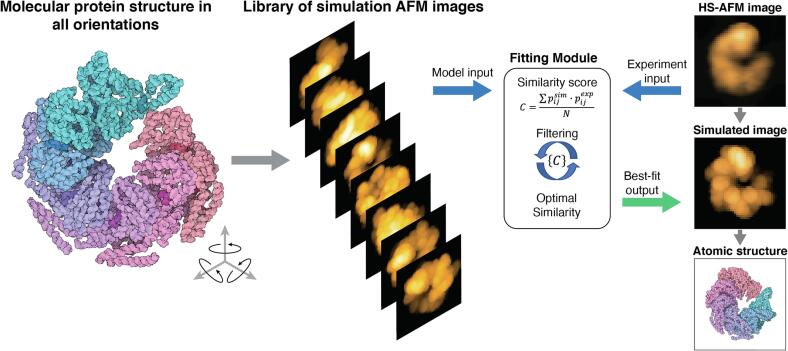
Automatized fitting to infer atomistic structures from resolution-limited experimental imaging. For a given PDB structure, simulation AFM can compute surface topographies of any possible molecular orientation, generating a library of simulated AFM images. The BioAFMviewer fitting module identifies the molecular structure whose simulated image matches best with an experimental target AFM image, quantified by an optimal image similarity score. A two-layered search strategy consisting of global unbiased sampling and refined iterative search ensures efficient automatized fitting taking typically only a few minutes on a laptop computer. Therefore, simulation AFM and automatized fitting within the BioAFMviewer allow to identify the atomistic structure behind measured resolution-limited AFM topographies.

Integrated into the BioAFMviewer is a module which employs simulation AFM for automatized rigid-body fitting of available biomolecular structures to experimental AFM images. Thus, it becomes possible to identify the 3D atomistic structure behind measured AFM topographies (Fig. 2). This opens the opportunity to employ the enormous amount of available high-resolution structural data to facilitate the interpretation and understanding of resolution-limited scanning experiments.

### 2.3 Applications to experimental AFM images

**Fig. 3:**
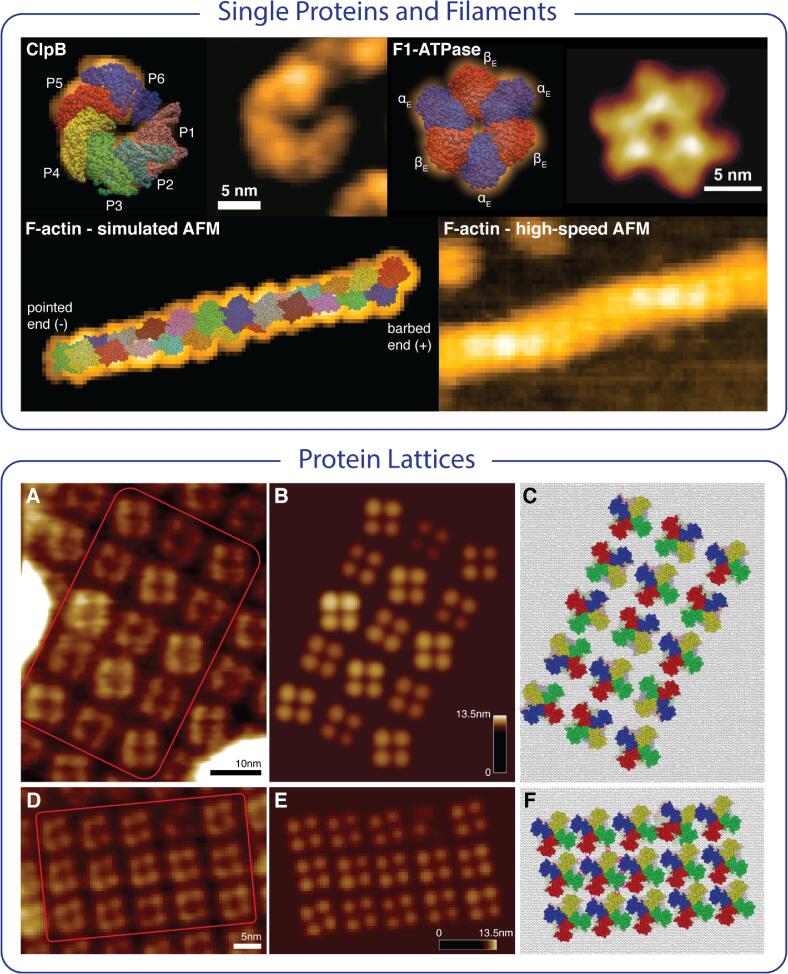
Applications to experimental AFM images. Automatized fitting was applied to obtain the 3D atomistic structures from experimental topographies ranging from single proteins and filaments to assemblies of 2D protein lattices. For the protein machines ClpB and rotor-less F1-ATPase the arrangement of functional domains can be disambiguated, and, even their nucleotide state can be inferred from HS-AFM topographies. Fitting an atomic model consisting of 24 actin subunits to a HS-AFM image of an actin filament demonstrates efficient fitting even for large proteins and allows to infer filament polarity. Application to HS-AFM imaging of lattices formed by protein channels in lipid bilayers allows to better understand their ligand-dependent activation mechanism. The atomic reconstruction from experimental imaging channels in their resting state versus activated state arrangement reveals differences of functional importance. While in their resting state configuration transmembrane domains of adjacent channels can form tight contact interfaces resulting in a quasi-static symmetric lattice, domains motions are much less restricted for channels in the ligand-induced activated state having a less ordered arrangement with larger separation of neighboring channels. Such information could not be deduced from AFM topographies since any transmembrane domain motions cannot be detected by the scanning tip. The ClpB HS-AFM image is adopted from (Uchihashi et al., 2018) and that of F1-ATPase is from (Ando et al., 2014).

A plethora of applications, ranging from single molecular machines, protein filaments, to large-scale assemblies of protein lattices, demonstrate how the full atomistic information obtained from simulation AFM and efficient automatized fitting advances the molecular understanding beyond original resolution-limited topographic AFM imaging (Fig. 3).

### 2.4 Topography analysis tools for quantitative feature assignment

**Fig. 4:**
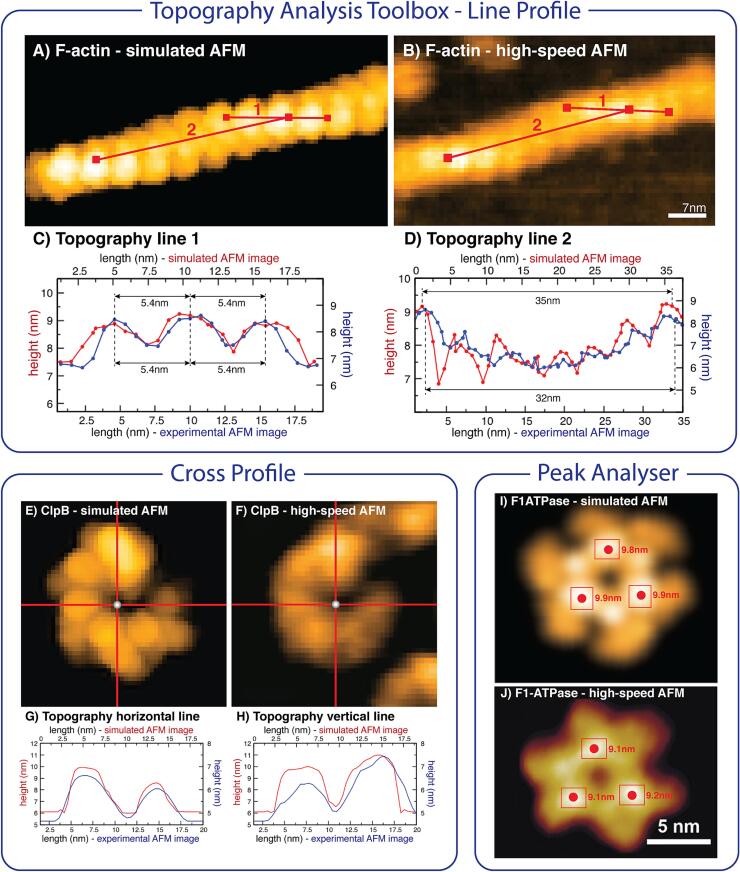
Topography Analysis Tools. A variety of tools allow quantitative topography analysis to validate experimental observations by employing simulation AFM and automatized fitting. Several applications where simulated topographies obtained after fitting are compared with measured images demonstrate a remarkable agreement. In case of the actin filament the spatial separation between individual monomers as well as the half-helical pitch of the periodic arrangement compare well. For the ClpB and rotor-less F1-ATPase protein machines height profiles show a well agreement of relative surface heights in simulated and HS-AFM images. Such applications demonstrate that by more detailed analysis, simulation AFM allows validation of experimental observations and quantitative feature assignment with measured topographies. A noteworthy observation is that absolute heights determined in simulated AFM topographies are systematically larger compared to measured topographies. This is due to the approximation of non-elastic tip-scanning with a rigid atomic model of the biomolecular structure, whereas experimental tip-scanning probes a deformable structure and is also invasive. Nonetheless, the agreement of relative heights of the scanned protein topographies is remarkable.

The BioAFMviewer contains a topography analysis toolbox which allows comparison of simulated and measured topographies. Several applications demonstrate that by a more detailed analysis, simulation AFM allows the validation of measurements and quantitative feature assignment within resolution-limited measured topographies (Fig. 4).

### 2.5 Simulation AFM experiments

**Fig. 5:**
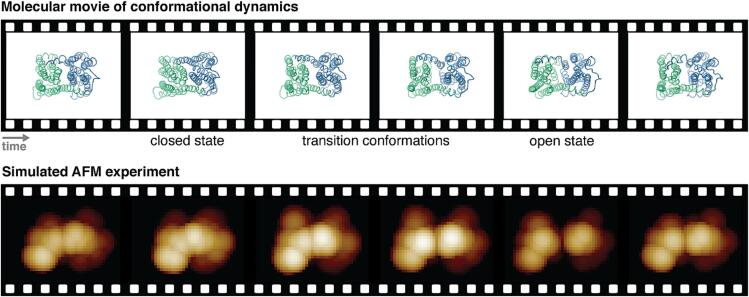
Simulation AFM experiments. Available static structures can be employed in multi-scale molecular modelling to explore non-equilibrium functional conformational dynamics in proteins, thus providing high-resolution molecular movies of their operation cycles. In the BioAFMviewer simulation AFM of such molecular movies can be performed to produce computational AFM experiments. The top row shows consecutive snapshots from a molecular movie of a transmembrane transporter, visualizing conformational motions of the opening-closing transition which underlies the operation cycle. The molecular movie was obtained employing elastic-network based Brownian dynamics simulations (Oranella et al., 2016). The bottom row displays the corresponding topographic images of the simulated experiment in the same perspective.

Applications of simulation AFM go beyond fitting of equilibrium data to infer atomistic structures from AFM imaging. Molecular movies of biomolecular conformational dynamics obtained from simulations can be processed in the BioAFMviewer to generate simulated AFM experiments (Fig. 5). Simulated AFM experiments can in principle complement real HS-AFM experiments to extract high-resolution 4D structural dynamics from AFM data. Eventually, the ambitious goal in future applications is to employ molecular modeling to reconstruct a high-resolution molecular movie with atomistic information from a HS-AFM topographic movie. Such methods require fitting of modeling data one-by-one to individual images from the experimental movie, employing flexible (Niina et al., 2020, Dasgupta et al., 2021) or rigid-body fitting methods (Niina et al., 2021, Amyot et al., 2022). First attempts have been recently presented (Ogane et al., 2022).

## 3 Summary and outlook

Simulation AFM plays an increasingly important role to complement experimental AFM imaging. In fact, given the enormous amount of high-resolution protein structures already available from the Protein Data Bank, together with the rapidly increasing data from AlphaFold predictions, there is no excuse not to employ simulation AFM to facilitate the interpretation of resolution-limited AFM observations by inferring 3D atomistic information from measured topographies. The BioAFMviewer platform has become a standard tool used by the Bio-AFM community worldwide. The increasing number of applications evidence the importance of simulation AFM. Further software developments are actively pursued and closely coordinated by intense collaborations with experimental groups.

## Declaration of Competing Interest

The authors declare that they have no known competing financial interests or personal relationships that could have appeared to influence the work reported in this paper.

## Data Availability

Data will be made available on request.

## References

[b0005] Amyot R., Flechsig H. (2020). BioAFMviewer: an interactive interface for simulated AFM scanning of biomolecular structures and dynamics. PLoS Comput. Biol..

[b0010] Amyot R., Marchesi A., Franz C.M., Casuso I., Flechsig H. (2022). Simulation atomic force microscopy for atomic reconstruction of biomolecular structures from resolution-limited experimental images. PLoS Comput. Biol..

[b0015] Ando T. (2022). High-speed atomic force microscopy in biology. Springer, Berlin, Heidelberg..

[b0020] Ando T., Uchihashi T., Scheuring S. (2014). Filming biomolecular processes by high-speed atomic force microscopy. Chem. Rev..

[b0025] Beton J.G., Moorehead R., Helfmann L., Gray R., Hoogenboom B.W., Joseph A.P., Topf M., Pyne A.L.B. (2021). TopoStats – a program for automated tracing of biomolecules from AFM images. Methods.

[b0030] Biyani M., Yasuda K., Isogai Y., Okamoto Y., Weilin W., Kodera N., Flechsig H., Sakaki T., Nakajima M., Biyani M. (2022). Novel DNA aptamer for CYP24A1 inhibition with enhanced antiproliferative activity in cancer cells. ACS Appl. Mater. Interfaces.

[b0040] Chaves RC, Pellequer J-L. 2013. DockAFM: benchmarking protein structures by docking under AFM topographs. Bioinformatics 29:3230-3231. doi: 10.1093/bioinformatics/btt561. 10.1093/bioinformatics/btt561 PMC5994945 24078683

[b0045] Chaves R.C., Teulon J.-M., Odorico M., Parot P., Chen S.-W., Pellequer J.-L. (2013). Conformational dynamics of individual antibodies using computational docking and AFM. J. Mol. Recognit..

[b0050] Dasgupta B., Miyashita O., Tama F. (2020). Reconstruction of low-resolution molecular structures from simulated AFM force microscopy images. Biochim. Biophys. Acta—Gen Subj..

[b0055] Dasgupta B., Miyashita O., Uchihashi T., Tama F. (2021). Reconstruction of three-dimensional conformations of bacterial ClpB from high-speed atomic-force-microscopy images. Front. Mol. Biosci..

[b0060] Flechsig H., Mikhailov A.S. (2019). Simple mechanics of protein machines. J. R. Soc. Interface.

[b0065] Heath G.R., Kots E., Robertson J.L., Lansky S., Khelashvili G., Weinstein H., Scheuring S. (2021). Localization atomic force microscopy. Nature.

[b0070] Ishimura R., El-Gowily A.H., Noshiro D., Komatsu-Hirota S., Ono Y., Shindo M., Hatta T., Abe M., Uemura T., Lee-Okada H.-C., Mohamed T.M., Yokomizo T., Ueno T., Sakimura K., Natsume T., Sorimachi H., Inada T., Waguri S., Noda N.N., Komatsu M. (2022). The UFM1 system regulates ER-phagy through the ufmylation of CYB5R3. Nat. Commun..

[b0075] Jumper J., Evans R., Pritzel A., Green T., Figurnov M., Ronneberger O., Tunyasuvunakool K., Bates R., Žídek A., Potapenko A., Bridgland A., Meyer C., Kohl S.A.A., Ballard A.J., Cowie A., Romera-Paredes B., Nikolov S., Jain R., Adler J., Back T., Petersen S., Reiman D., Clancy E., Zielinski M., Steinegger M., Pacholska M., Berghammer T., Bodenstein S., Silver D., Vinyals O., Senior A.W., Kavukcuoglu K., Kohli P., Hassabis D. (2021). Highly accurate protein structure prediction with AlphaFold. Nature.

[b0080] Kenzaki H., Koga N., Hori N., Kanada R., Li W., Okazaki K.-I., Yao X.-Q., Takada S. (2011). CafeMol: a coarse-grained biomolecular simulator for simulating proteins at work. J. Chem. Theory Comput..

[b0085] Lim K., Nishide G., Yoshida T., Watanabe‐Nakayama T., Kobayashi A., Hazawa M., Hanayama R., Ando T., Wong R.W. (2021). Millisecond dynamic of SARS-CoV-2 spike and its interaction with ACE2 receptor and small extracellular vesicles. J Extracell. Vesicles.

[b0090] Marchesi A., Umeda K., Komekawa T., Matsubara T., Flechsig H., Ando T., Watanabe S., Kodera N., Franz C.M. (2021). An ultra-wide scanner for large-area high-speed atomic force microscopy with megapixel resolution. Sci. Rep..

[b0095] Matusovsky O, Mansson A, Rassier DE. 2022. Cooperativity of myosin II motors in the non-regulated and regulated thin filaments investigated with high-speed AFM. 10.1085/jgp.202213190 PMC9859764 36633585

[b0100] Moretto L., Usaj M., Matusovsky O., Rassier D.E., Friedman R., Mansson A. (2022). Multistep orthophosphate release tunes actomyosin energy transduction. Nat. Commun..

[b0105] Müller D.J., Dufrêne Y.F. (2008). Atomic force microscopy as a multifunctional molecular toolbox in nanobiotechnology. Nature Nanotechnol..

[b0110] Niina T., Fuchigami S., Takada S. (2020). Flexible fitting of biomolecular structures to atomic force microscopy images via biased molecular simulations. J. Chem. Theory Comput..

[b0115] Niina T., Matsunaga Y., Takada S. (2021). Rigid-body fitting to atomic force microscopy images for inferring probe shape and biomolecular structure. PLoS Comput. Biol..

[b0120] Ogane T., Noshiro D., Ando T., Yamashita A., Sugita Y., Matsunaga Y. (2022). Development of hidden Markov modeling for molecular orientations and structure estimation from high-speed atomic force microscopy time-series images. PLoS Comput. Biol..

[b0125] Oranella L., Yoluk O., Carrilo O., Orozco M., Lidahl E. (2016). Prediction and validation of protein intermediate states from structurally rich ensembles and coarse-grained simulations. Nat. Commun..

[b0130] Poma A.B., Cieplak M., Theodorakis P.E. (2017). Combining the MARTINI and structure-based coarse-grained approaches for the molecular dynamics studies of conformational transitions in proteins. J. Chem. Theor. Comput..

[b0135] Scheuring S., Boudier T., Sturgis J.N. (2007). From high-resolution AFM topographs to atomic models of supramolecular assemblies. J Struct Biol..

[b0140] Schindelin J., Arganda-Carreras I., Frise E., Kaynig V., Longair M., Pietzsch T. (2012). Fiji: an open-source source platform for biological-image analysis. Nat Methods.

[b0145] Takeda K, Muro I, Kobayashi F, Flechsig H, Kodera N, Ando T, et al. 2022. Structural dynamics of E6AP E3 ligase HECT domain and involvement of flexible hinge loop in ubiquitin chain synthesis mechanism. Preprint: bioRxiv. doi: 10.1101/2022.11.18.516873. 10.1021/acs.nanolett.3c04150 PMC10755755 38055898

[b0150] Trinh M-H, Odorico M, Pique ME, Teulon J-M, Roberts VA, Ten Eyck LF, et al. 2012. Computational reconstruction of multidomain proteins using atomic force microscopy data. Structure 10:113-120. doi: 10.1016/j.str.2011.10.023. 10.1016/j.str.2011.10.023 PMC3264848 22244760

[b0155] Uchihashi T., Iino R., Ando T., Noji H. (2011). High-speed atomic force microscopy reveals rotary catalysis of rotorless F1-ATPase. Science.

[b0160] Uchihashi T., Watanabe Y.-H., Nakazaki Y., Yamasaki T., Watanabe H., Maruno T., Ishii K., Uchiyama S., Song C., Murata K., Iino R., Ando T. (2018). Dynamic structural states of ClpB involved in its disaggregation function. Nat. Commun..

[b0035] wwPDBconsortium. Protein data bank: the single global archive for 3D macromolecular structure data. 2019. Nucleic Acids Res. 47: D520–D528. doi: 10.1093/nar/gky949. 10.1093/nar/gky949 PMC6324056 30357364

[b0165] Yamada R., Trang T.N., Flechsig H., Takeda T., Kodera N., Konno H. (2022). Importance of annexin V N-terminus for 2D crystal formation and quick purification protocol of recombinant annexin V. PLoS ONE.

